# Bayesian Data Assimilation of Temperature Dependence of Solid–Liquid Interfacial Properties of Nickel

**DOI:** 10.3390/nano11092308

**Published:** 2021-09-06

**Authors:** Yuhi Nagatsuma, Munekazu Ohno, Tomohiro Takaki, Yasushi Shibuta

**Affiliations:** 1 Department of Materials Engineering, The University of Tokyo, Tokyo 113-8656, Japan; nagatsuma-yuhi565@g.ecc.u-tokyo.ac.jp; 2 Division of Materials Science and Engineering, Faculty of Engineering, Hokkaido University, Sapporo 060-8628, Japan; mohno@eng.hokudai.ac.jp; 3 Faculty of Mechanical Engineering, Kyoto Institute of Technology, Kyoto 606-8585, Japan; takaki@kit.ac.jp

**Keywords:** data assimilation, ensemble Kalman filter, molecular dynamics simulation, phase-field model, solid–liquid interface, Bayesian inference

## Abstract

Temperature dependence of solid–liquid interfacial properties during crystal growth in nickel was investigated by ensemble Kalman filter (EnKF)-based data assimilation, in which the phase-field simulation was combined with atomic configurations of molecular dynamics (MD) simulation. Negative temperature dependence was found in the solid–liquid interfacial energy, the kinetic coefficient, and their anisotropy parameters from simultaneous estimation of four parameters. On the other hand, it is difficult to obtain a concrete value for the anisotropy parameter of solid–liquid interfacial energy since this factor is less influential for the MD simulation of crystal growth at high undercooling temperatures. The present study is significant in shedding light on the high potential of Bayesian data assimilation as a novel methodology of parameter estimation of practical materials an out of equilibrium condition.

## 1. Introduction

Phase-field simulation [1,2,3,4] is a powerful tool to deal with free boundary problems and is widely applied to various investigations of microstructural evolution, including solidification [1,2,3,4,5,6], grain growth [7,8,9], recrystallization [10], and solid-state phase transformation [11,12]. However, necessary parameters are incompletely available to reproduce morphological transition with high precision in most cases because of difficulties with experimental measurements. In particular, it is not straightforward to measure solid–liquid interfacial properties in spite of many efforts over many years [13,14,15]. Therefore, various molecular dynamics (MD) simulations have contributed to the estimation of solid–liquid interfacial energy and mobility [16,17,18,19,20,21]. The capillary fluctuation method (CFM) [18,19] is the most popular technique for estimation of solid–liquid interfacial energy, including its anisotropy, and this technique has been applied to various metals and alloys [22,23,24,25,26]. Moreover, classical nucleation theory (CNT)-based techniques are often employed to estimate the solid–liquid interfacial energy [20,27,28]. Interfacial parameters estimated from MD simulations are widely employed for phase-field simulations in the framework of multi-scale modeling [29,30].

However, interfacial parameters estimated from MD simulations are largely limited to those of static interfaces in equilibrium conditions. For example, the capillary fluctuation method is mostly applicable at melting point [18,19,28], where the solid–liquid interface does not propagate. Moreover, temperature dependence of interfacial properties is mostly deduced in the CNT-based method under the assumption of temperature dependence of thermodynamic parameters [27,28]. Therefore, it is not straightforward to discuss the change in solid–liquid interfacial energy out of the equilibrium condition. Specifically, the temperature dependence of the solid–liquid interfacial energy is still under discussion [31,32,33,34,35,36,37,38,39]. Several theoretical and computational studies have reported that the solid–liquid interface increased with increasing temperature (i.e., the positive temperature dependence) [31,32,33,34,35,36,37,38], whereas other literature has indicated that the temperature dependence of solid–liquid interfacial energy was not monotonic [39,40]. Different temperature dependencies for the interfacial energy of an unstable equilibrium (i.e., for nuclei) have been theoretically predicted [41]. Recently, negative temperature dependence of solid–liquid interfacial energy for Cu-Zr and Al-Sm alloys was reported [42,43,44]. Moreover, it has been reported that temperature dependence is affected by the definition of simulation parameters [45]. Therefore, it is essential to establish a reliable methodology to define the solid–liquid interfacial properties.

Recently, we proposed a new approach for the estimation of interfacial properties out of equilibrium (i.e., for moving interface below the melting point) on the basis of the Bayesian inference theory [46] in the framework of data assimilation. Data assimilation [47] is a mathematical description for combining numerical simulations with observation data to estimate states and/or parameters of the system, as well as to enhance simulation accuracy [46]. Data assimilation had achieved great success, mainly in geophysics, in the early stage [48]. Recently, data assimilation has appeared in the field of materials science [49,50,51,52,53,54], including grain growth [49,50], solidification [51,52], welding [53], and fatigue crack propagation [54]. These studies shed light on the high potential of the data assimilation approach for the estimation of unmeasurable states and/or parameters during materials processing. In our previous study [46], solid–liquid interfacial energy, interfacial mobility, and anisotropy parameters of body-centered cubic (bcc)-Fe were simultaneously estimated through a data assimilation approach, in which microstructural data obtained from the MD simulation were employed as observation data in conjunction with the phase-field model as a simulation model. Although this approach is promising for parameter estimation from observation data, further studies are required to confirm its versatility. To this end, we expand this method to include the solid–liquid interfacial properties of nickel as the most practical face-centered cubic (fcc) metals in the present study. Specifically, we newly propose a method of utilizing multiple experiments of parameter estimation while decreasing the target parameters to find less influential factors in the observation data without any prior knowledge.

## 2. Phase-Field Model

In the phase-field model, morphology of microstructure is defined by spatial distribution of the phase-field variable, *ϕ*. Here, *ϕ* = 1 and *ϕ* = −1 represent solids and liquids, respectively, and the solid–liquid interface is defined by the continuous change between −1 and 1. In this study, a phase-field model for the isothermal solidification of pure metal [55] is employed following our previous study [46], which is described as follows:

(1) $$\tau \left(n\right)\frac{\partial \phi }{\partial t}=\nabla \left[W{\left(n\right)}^{2}\nabla \phi \right]+\underset{i=x,y}{\sum }\partial_{i}\left({\left|\nabla \phi \right|}^{2}W\left(n\right)\frac{\partial W\left(n\right)}{\partial \left(\partial_{i}\phi \right)}\right)+\phi -\phi^{3}-\lambda {\left(1-\phi^{2}\right)}^{2}u_{int}$$

(2) $$W\left(n\right)=W_{0}a_{c}\left(n\right)$$

(3) $$\tau \left(n\right)=\frac{W_{0}^{2}}{d_{0}}\beta_{0}a_{c}\left(n\right)a_{k}\left(n\right)$$

Here, λ = *a*_1_*W*_0_/*d*_0_ is the coupling constant with *a*_1_ = 5√2/8, *W*_0_ is the interface thickness, and *d*_0_ is the capillary length represented by *d*_0_ = *σ*_0_ (*T_m_c_p_*/Δ*H*^2^). *β*_0_ is the kinetic coefficient. *u_int_* is the dimensionless undercooling at the interface defined as

(4) $$u_{int}=\frac{\Delta T}{\Delta H/c_{p}}$$

where ∆*T* is the undercooling temperature, ∆*H* is the latent heat, and *c_p_* is the specific heat. *τ* (**n**) and *W* (**n**) are relaxation time and interface width, respectively. *a_c_* (**n**) and *a_k_* (**n**) are functions based on the interface orientation, respectively, and are expressed as follows.

(5) $$a_{c}\left(n\right)=\left(1-3\varepsilon_{c}\right)\left(1+\frac{4\varepsilon_{c}}{1-3\varepsilon_{c}}\left(n_{x}^{4}+n_{y}^{4}\right)\right)$$

(6) $$a_{k}\left(n\right)=\left(1+3\varepsilon_{k}\right)\left(1-\frac{4\varepsilon_{k}}{1+3\varepsilon_{k}}\left(n_{x}^{4}+n_{y}^{4}\right)\right)$$

*ε_c_* and *ε_k_* represent the strength of anisotropy of interfacial energy and the kinetic coefficient. (*n_x_*, *n_y_*) is the normal vector at the interface obtained by

(7) $$n=\left(n_{x},n_{y}\right)=-\nabla \phi =\left(-\frac{\partial \phi }{\partial x},-\frac{\partial \phi }{\partial y}\right)$$

## 3. Data Assimilation Based on Ensemble Kalman Filter

### 3.1. State Vector and System Model

Data assimilation is a method to combine a simulation model with observation data to estimate states and/or parameters of the system [47]. In the simulation model, solution of the governing equation is deterministically derived once initial and boundary conditions are given. However, it is, in general, impossible for the simulation to reproduce phenomena in nature precisely since there is a discrepancy between simulation results and true dynamics due to the incompleteness of the simulation model, fluctuations in the phenomenon, unknown boundary conditions, and so on. Therefore, uncertainty of time evolution of states is considered by introducing state variables with a probability distribution, which is called a system model. That is the basic concept of the data assimilation.

In the simulation, time evolution of a system is calculated by discretizing the governing equation in time and space in general. Physical quantities are represented by values at the representative points (mostly grid points). A column vector consisting of all representative values $\epsilon_{i,j}$ on the grid point (*i*, *j*) at time *t* is called state vector ***x****_t_*:

(8) $$x_{t}\equiv \left(\begin{array}{c} \epsilon_{0,0}\\ \epsilon_{0,1}\\ \vdots \\ \epsilon_{i,j}\\ \vdots \\ \epsilon_{m,n} \end{array}\right)$$

Each element of the state vector is called a state variable. A system model, which derives the state vector ***x****_t_* at time *t* from the state vector ***x**_t_*_–1_ at time *t* − 1, is defined as

(9) $$x_{t}=f_{t}\left(x_{t-1},\;v_{t}\right)$$

where ***f****_t_* is the simulation model at time *t* and ***v****_t_* is the system noise representing the imperfectness of simulation model. The observation model, which represents the relationship between observation data ***y****_t_* and the state vector ***x****_t_* at time *t*, is defined as

(10) $$y_{t}=h_{t}\left(x_{t}\right)+w_{t}$$

Observation noise ***w****_t_* includes measurement error and imperfectness of the simulation model.

### 3.2. Ensemble Kalman Filter (EnKF)

**Figure 1:**
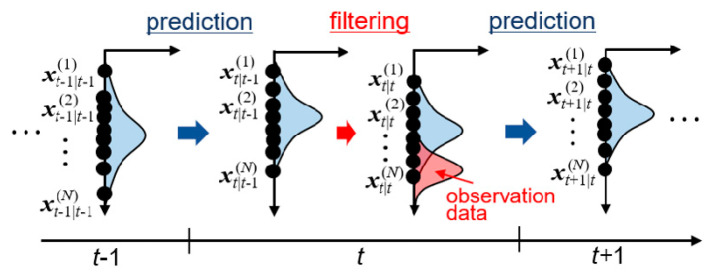
Schematic image of prediction and filtering processes at time *t* by ensemble Kalman filter (EnKF).

In data assimilation, state variables follow a probability density function. State variables are iteratively updated by integrating observation data into existing probability density functions based on the Bayesian inference approach [47]. This procedure is called filtering. There are various types of filtering approaches, including Kalman filter [56], ensemble Kalman filter (EnKF) [57,58], and particle filter [59]. In the present study, EnKF was employed. In the EnKF, many simulations with different parameters are performed in parallel, which represents the probability distribution function. Figure 1 shows schematic image of the calculation procedure of EnKF, which consists of cycles of prediction and filtering steps. Multiple simulations (called ensembles) are independently performed in the prediction step to obtain predictive state variables and parameters, which are then corrected based on the observation data. Mathematical expression of the EnKF is summarized in Appendix A. Expected values of parameters are obtained from the ensemble average at each filtering step as sequential data with respect to time. The estimated value of each parameter is then obtained by a time average of the expectation value in the time period after the convergence.

### 3.3. Calculation Procedure of Data Assimilation

In this study, kinetic coefficient *β*_0_, interface energy *σ*_0_, and their anisotropy parameters *ε_k_* and *ε_c_* during the growth of a single crystal under isothermal conditions were estimated by the EnKF procedure. The time evolution equation of the phase-field method (Equation (1)) was used as *f_t_* in the system model (Equation (A1)), which describes the time evolution of state vectors from *t* − 1 to *t*. Equation (1) was discretized in a standard finite different scheme with second order accuracy in the space and it was solved in an explicit Euler scheme. The calculation system was divided into 100 × 100 grid points. State variable vector ***x****_t_* and observation vector ***y****_t_* are given as:

(11) $$x_{t}=\left\{\begin{array}{c} \phi_{1,1}\\ \phi_{1,2}\\ \phi_{1,3}\\ \ldots \\ \phi_{100,100}\\ \beta_{0}\\ \varepsilon_{k}\\ \sigma_{0}\\ \varepsilon_{c} \end{array}\right\}$$

(12) $$y_{t}=\left\{\begin{array}{c} \phi_{1,1}\\ \phi_{1,2}\\ \phi_{1,3}\\ \vdots \\ \phi_{100,100} \end{array}\right\}$$

A total of 10,004 variables, including phase-field variables *ϕ_i_*_,*j*_ at all lattice points (*i*, *j*) and four parameters to be estimated (*β*_0_, *σ*_0_, *ε*_k_, *ε*_c_), were used as state variables in state variable vectors. The phase-field variables at each grid point in the observation data were used as the observation variables in the observation vector. Table 1 and Table 2 show the parameters used for the phase-field simulation and for the EnKF data assimilation, respectively. We created 100 phase-field simulations using independent state vectors and optimized the simulations based on the observed data by alternately executing prediction by the system model and filtering by Equation (A6). Equation (A1) was used as a system model, where Equation (1) was employed as a nonlinear operator *f_t_*, and Equation (A2) was used as an observation model. The observation noise ***w****_t_* was set as a random number vector generated from Gaussian distribution according to covariance matrix *R_ϕ_* which is an identity matrix in the shape of 10,000 × 10,000. Observation matrix ***H****_t_* in the observation model is given as:

(13) $$H_{t}=\left[\begin{array}{ccccccc} 1 & 0 & \cdots & 0 & 0 & \cdots & 0\\ 0 & 1 & \cdots & 0 & \vdots & \cdots & \vdots \\ \vdots & \vdots & \ddots & \vdots & \vdots & \cdots & \vdots \\ 0 & 0 & \cdots & 1 & 0 & \cdots & 0 \end{array}\right]$$

where the dimension of the matrix is 10,000 × 10,004. In this study, atomic configurations from MD simulations of the growth of a single crystal of nickel were employed as observation data, the preparation of which is described in the next section.

## 4. Molecular Dynamics Simulation for Observation Data

Prior to the data assimilation by EnKF, observation data were prepared by MD simulation. MD simulation was performed using large-scale atomic/molecular massively parallel simulator (LAMMPS) [61]. An embedded atom method (EAM) potential fitted by Purja Pun and Mishin [62,63] was employed to obtain the interatomic potential for nickel. Representative properties are listed in Supplementary Materials. In the present study, nickel was employed as a representative face-centered cubic (fcc) metal. It is known that interfacial anisotropy of fcc metals is generally larger than that of body-centered cubic (bcc) metals [64]. Velocity–Verlet method was used to integrate the classical equation of motion with a time step of 1.0 fs. Nose–Hoover thermostat and barostat [65,66] were employed to control temperature and pressure. Note that there exists a large temperature distribution in the calculation system during crystal growth due to the release of latent heat with a conventional thermostat [46,67], even though average temperature of the system is steadily controlled at the target temperature. Therefore, Langevin thermostat [68] was also employed to keep the temperature uniform over all areas of the system, which is essential for a precise comparison with the isothermal phase-field model.

**Figure 2:**
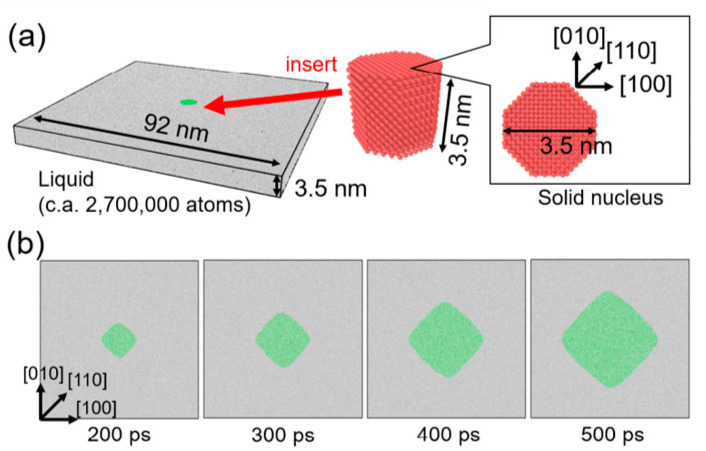
Molecular dynamics simulation of the growth of a single crystal of nickel from an undercooled melt for observation data for data assimilation. (**a**) Initial configuration of simulation system. (**b**) Snapshots of atomic configuration during the crystal growth from the undercooled melt of Δ*T* = 200 K. Green and white atoms represent solid and liquid atoms, respectively, which were identified by polyhedral template matching.

Figure 2a shows the initial configuration of the calculation system for MD simulation, which was prepared as follows. The liquid structure was prepared by heating an fcc crystal of nickel consisting of 260 × 260 × 10 unit cells (2,704,000 atoms) at 2000 K with the canonical (i.e., the number of atoms, volume, and temperature constant) ensemble for 10 ps. Separately, a solid nucleus was prepared as an octagonal cutout from the fcc crystal with four {100} and four {110} facets. The solid nucleus was then inserted into the liquid structure while omitting all liquid atoms located within 2.5 Å from a solid atom to avoid unexpected proximity between liquid and solid atoms at the interface. Energy minimization was performed for the combined structure. The prepared structure was then relaxed at 1455, 1480, 1505, and 1530 K for 800 ps with the isobaric–isothermal (i.e., the number of atoms, pressure, and temperature constant) ensemble and growth behavior of the solid nucleus was investigated. Periodic boundary condition was employed in all directions. Note that the melting point of Ni of this EAM potential was approximately 1680 K [60], which was estimated by the convergence temperature technique [69,70]. That is, the temperature 1455, 1480, 1505, and 1530 K corresponds to the undercooling temperature Δ*T* = 225, 200, 175, and 150 K. Atomic configurations from MD simulation were analyzed by polyhedral template matching (PTM) [71] implemented in the Open Visualization Tool (OVITO) [72] to identify local atomic structures (i.e., solid or liquid).

Figure 2b shows snapshots of atomic configuration during growth of the solid nucleus from the undercooled melt of Δ*T* = 200 K. The nucleus grew preferentially in <100> directions and it became a rhombic-like structure, which means that fourfold symmetry appeared. The obtained atomic configuration was not suitable for the observation data for data assimilation with phase-field simulation. Therefore, atomic configuration from the MD simulation was converted into a phase-field profile in line with our previous studies [73,74]. A cross-section (90 × 90 nm^2^) of the MD simulation cell was divided into two-dimensional difference grids of 100 × 100. After assigning all atoms in the closest grid, the majority of local atom configurations (i.e., solid or liquid) for assigned atoms were employed as the phase-field variables of each grid point (solid: 1, liquid −1). Since this voxel structure had no interfacial thickness, it was relaxed by solving the phase-field equation without the curvature effect [73] to obtain the phase-field profile with diffuse interface. This conversion procedure was carried out for the time series of atomic configuration of MD simulation with 10 ps interval. Obtained phase-field profiles were employed as the observation data for data assimilation. The observation data between 300 and 600 ps at 10 ps interval were used in the data assimilation in following the sections to avoid the initial relaxation period of solid nucleus in the MD simulation.

## 5. Results and Discussion

**Figure 3:**
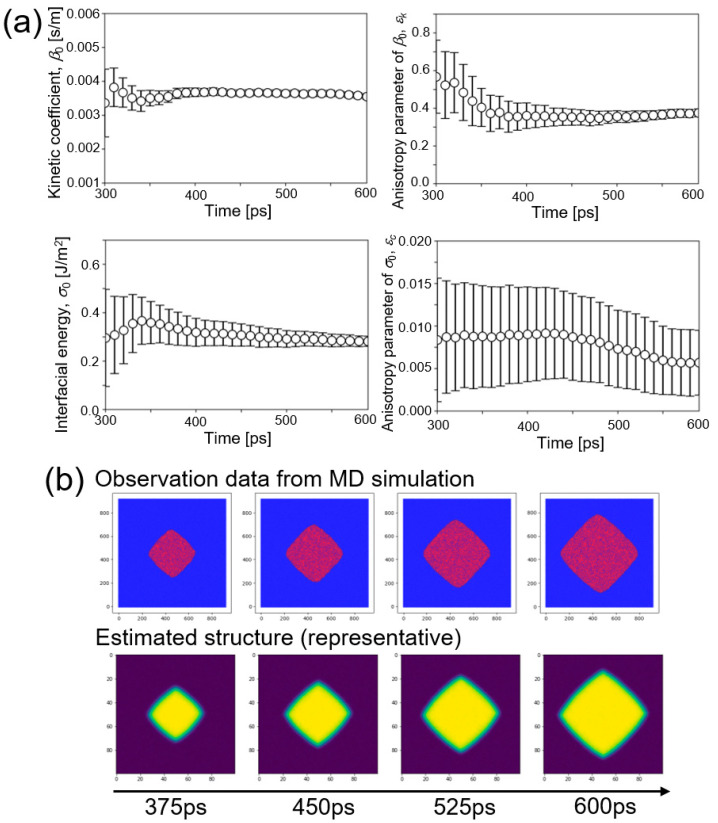
Estimation of four parameters (kinetic coefficient *β*_0_, interfacial energy *σ*_0_, and their anisotropy parameters *ε_k_* and *ε_c_*) using observation data of molecular dynamics (MD) simulation at 1480 K. (**a**) Time changes of the estimated values of four parameters *β*_0_, *ε_k_*, *σ*_0_, and *ε_c_*. (**b**) Snapshots of observation data from the MD simulation and representative results of the estimated structure.

Now, parameter estimation of *β*_0_, *σ*_0_, *ε_k_*, and *ε_c_* from the dataset of MD simulation at 1480 K was performed. Figure 3a shows time change of the estimated values of four parameters. Estimated values of three parameters, *β*_0_, *ε_k_*, and *σ*_0_ converged to certain values with decreasing the variance. On the other hand, variance of the estimated value of *ε_c_* did not decrease during the estimation although the estimated value itself came close to a certain value. It was expected that accuracy of estimation of *ε_c_* was lower than those of the other parameters.

Figure 3b shows snapshots of observation data from the MD simulation and representative results of the estimated structure. The crystal shape in the observation data and that of the representative ensemble member were in good agreement. The same procedure was performed for other datasets of MD simulations at 1455, 1480, and 1505 K. These results are summarized in the Supplementary Materials. In general, results for the other temperatures agreed with that of 1480 K. That is, estimated values of three parameters, *β*_0_, *ε_k_*, and *σ*_0_ converged to certain values with decreasing variance, while accuracy of estimation of *ε_c_* was again low compared to the other parameters.

**Figure 4:**
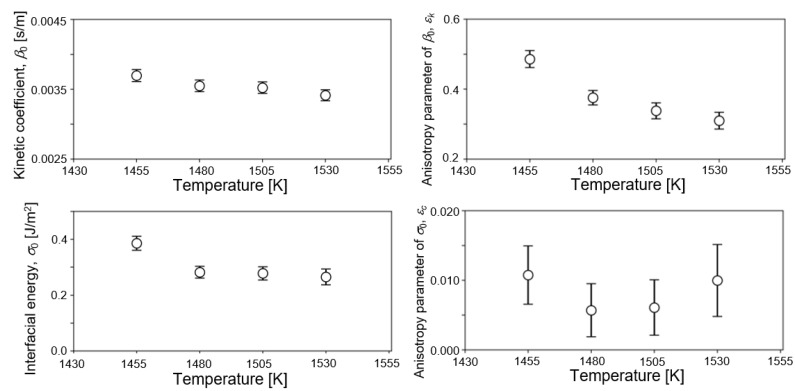
Temperature dependence of the estimated values of four parameters from observed data of 1455, 1480, 1505, and 1530 K. Estimated values on the last filtering step are plotted in the figure.

Figure 4 shows the temperature dependence of estimated values of four parameters from observed data of 1455, 1480, 1505, and 1530 K. Estimated values of the last filtering step are plotted in the figures. Estimated values of *β*_0_, *ε_k_*, and *σ*_0_ decreased with increasing temperature. The negative temperature dependence of *σ*_0_ agreed with our previous estimation of the solid–liquid interfacial energy of bcc-Fe by EnKF [46] and some reports in the literature [42,43,44]. Bayesian inference derived the most probable values of solid–liquid interfacial energy at various temperatures from the results of the MD simulation without any prior knowledge. It is guaranteed that the phase-field model employed in this study reproduces the Gibbs–Thomson effect properly. Therefore, the parameters derived in this study were appropriately within the range where the Gibbs–Thomson effect is valid. One possible reason of discrepancy from some studies of positive temperature dependence may be due to the effect of interface curvature [37,41]. However, it was difficult to find the physical origin of negative temperature dependence directly from our result. The degree of temperature dependence of *β*_0_ was smaller than that of *σ*_0_, Incidentally, *β*_0_ was nearly independent of the temperature within the examined temperature range in our previous study for bcc-Fe [46]. Moreover, it was difficult to find a clear trend in the temperature dependence of *ε_c_* since the accuracy of estimation of *ε_c_* was lower, as described above. The estimated values of *σ*_0_ ranged from 0.27 to 0.38 J/m^2^, which basically overlapped experimental and theoretical values of *σ*_0_ for Ni at melting point (0.255 [13], 0.284 [75], 0.306 [76], and 0.325 J/m^2^ [14]). Regarding the kinetic coefficient, *β*_0_ took values between 0.0035 and 0.0037 s/m. *β*_0_ can be converted into the interfacial mobility *μ* by the following relation, *μ* = *c_p_*/*β*_0_Δ*H* [55]. Using the values of *c_p_* and Δ*H* in Table 1, *β*_0_ = 0.0035 m/s was converted into *μ* = 0.418 m/sK. This is within the range of reported values, 0.18–0.45 m/sK, which were derived from MD simulations with planar solid–liquid interfaces of Ni [77]. It is convincing that both the solid–liquid interfacial energy and interfacial mobility estimated in this study were consistent with previous reported values from various methodologies. On the other hand, temperature dependence of *ε_k_* took the opposite trend to our previous estimation of bcc-Fe, which was the positive temperature dependence. This difference might come from the difference in the strength of anisotropy. That is, a strong anisotropy appeared in the crystal structure of fcc-Ni in this study, whereas a weak anisotropy appeared in that of bcc-Fe structure [46]. Further study is needed to discuss the anisotropy in interfacial mobility.

As described above, the accuracy of estimation of *ε_c_* was low compared to the other parameters in the four-parameter estimation. Therefore, *ε_c_* was separately estimated while fixing the other parameters at estimated values of *β*_0_ = 0.00275 [s/m], *ε_k_* = 0.338, and *σ*_0_ = 0.277 [J/m^2^]. For this one parameter estimation, state variable vector and the observation matrix were modified as follows.

(14) $$x_{t}=\left\{\begin{array}{c} \phi_{1,1}\\ \phi_{1,2}\\ \phi_{1,3}\\ \ldots \\ \phi_{100,100}\\ \varepsilon_{c} \end{array}\right\}$$

**Figure 5:**
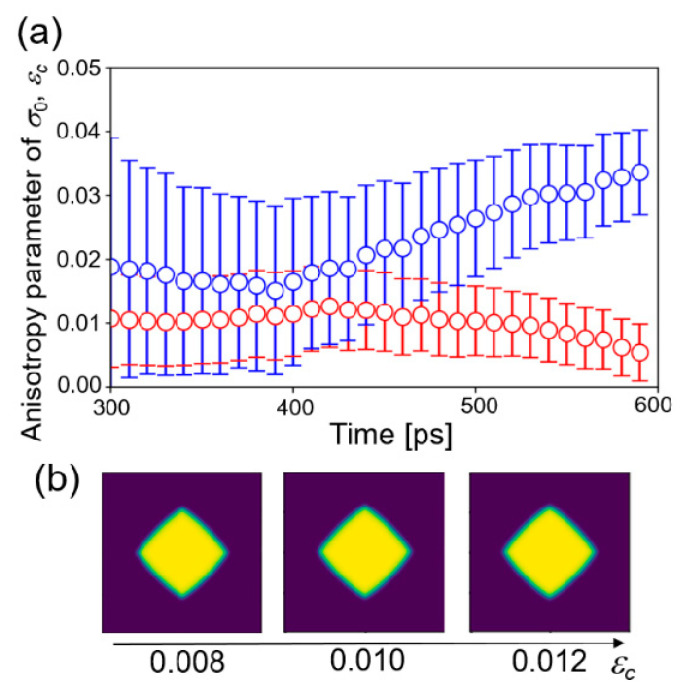
(**a**) Time changes of the estimated value of the anisotropy parameter *ε_c_* in one parameter estimation starting from two initial distributions. (**b**) Phase-field profiles from the phase-field simulations with fixed parameters.

Observation vector ***y****_t_* was the same as Equation (12). The observation matrix is given as:

(15) $$H_{t}=\left[\begin{array}{ccccc} 1 & 0 & \cdots & 0 & 0\\ 0 & 1 & \cdots & 0 & \vdots \\ \vdots & \vdots & \ddots & \vdots & \vdots \\ 0 & 0 & \cdots & 1 & 0 \end{array}\right]$$

where the dimension of the matrix is 10,000 × 10,001. Figure 5a shows the time change of the estimated values of *ε_c_* starting from different initial distributions at 1505 K. The other conditions were the same as those of the four-parameter estimation. Two estimations did not converge to the same value. That is, it was not successful in obtaining the converged value of the estimation for *ε_c_*, even from the procedure of one parameter estimation.

Furthermore, the effect of the anisotropy parameter on growth morphology of crystal structure was examined by a phase-field simulation with fixed parameters. Three parameters were fixed to the values estimated above at 1455 K and three values of *ε_c_* = 0.008, 0.010, and 0.012 were employed. The other parameters were the same as listed in Table 1. Figure 5b shows the phase-field profile after 30,000 step simulations. The morphologies of the structures did not change significantly with respect to *ε_c_*. Therefore, it is considered that the effect of anisotropy of solid–liquid interfacial energy on the crystal structure is very small under the condition of large undercooling temperature. In that condition, growth velocity is very fast and the anisotropy in interfacial mobility is dominant. In other words, it is difficult to estimate the parameters of a less influential factor in the framework of the present study. The anisotropy parameter *ε_c_* may be estimated when a near equilibrium structure of the crystal is employed as observation data, which will be investigated in the next step.

## 6. Conclusions

In the present study, temperature dependence of solid–liquid interfacial properties during the crystal growth was investigated by data assimilation with EnKF. It is the advantage of the methodology in this study that both the interfacial energy and mobility out of equilibrium condition could be estimated simultaneously from Bayesian inference. Negative temperature dependence was found in the solid–liquid interfacial energy, kinetic coefficient, and anisotropy of kinetic coefficient from simultaneous estimations of four parameters. However, the anisotropy parameter of the solid–liquid interfacial energy did not converge during the four-parameter estimation and it did not converge even in the subsequent single parameter estimation. Since the anisotropy parameter of the solid–liquid interfacial energy did not affect the morphology of the crystal in the phase-field simulation with fixed parameters, it is difficult to estimate the parameter for the less influential factors in the observation phenomena. In other words, we can find less influential factors in the observation data without any prior knowledge for target phenomena. In summary, it is significant that this study showed the high potential of data assimilation as a methodology of parameter estimation in the out of equilibrium condition. The overlap between atomistic and continuum simulations, which was achieved owing to recent progress in high-performance computing, creates new research concepts and fields. We call this cross-scale modeling, as an evolution from conventional multi-scale modeling [30].

## Figures and Tables

**Table 1:** Parameters for phase-field simulation.

Parameter	Symbol	Value
Grid size [m]	Δ*x*	9.0 × 10^−10^
Interface thickness [m]	*W*_0_	2.0Δ*x* = 1.8 × 10^−9^
Latent heat [J/m^3^]	Δ*H*	2.83966 × 10^9^ [60]
Constant pressure specific heat [J/(m^3^K)]	*c_p_*	4.1578 × 10^6^ [60]
Temperature [K]	*T*	1455, 1480, 1505, 1530
Time step [s]	*Δ* *t*	1.0 × 10^−14^

**Table 2:** Parameters for EnKF data assimilation.

Parameter	Symbol	Value
Ensemble number	*−*	100
Filtering interval [s]	*−*	1.0 × 10^−^^11^
Total time [s]	*−*	3.0 × 10^−^^10^
System noise of *ϕ*	$Q_{\phi }$	1.0 × 10^−^^3^
System noise of *β*_0_	$Q_{\beta_{0}}$	1.0 × 10^−^^6^
System noise of *ε_k_*	$Q_{\varepsilon_{k}}$	1.0 × 10^−^^4^
System noise of *σ*_0_	$Q_{\sigma_{0}}$	1.0 × 10^−^^4^
System noise of *ε_c_*	$Q_{\varepsilon_{c}}$	1.0 × 10^−^^5^
Observation noise of *ϕ*	$R_{\phi }$	1.0

## Data Availability

The data presented in this study are available on request from the corresponding author.

## Supplementary Materials

The following are available online at https://www.mdpi.com/article/10.3390/nano11092308/s1, Table S1. Representative properties of the EAM potential for Ni employed in this study. Figure S1: Estimation of four parameters (kinetic coefficient *β*_0_, interfacial energy *σ*_0_, and their anisotropy parameters, *ε_k_* and *ε_c_*) using observation data of molecular dynamics (MD) simulation at 1455 K. Figure S2: Estimation of four parameters (kinetic coefficient *β*_0_, interfacial energy *σ*_0_, and their anisotropy parameters, *ε_k_* and *ε_c_*) using observation data of molecular dynamics (MD) simulation at 1505 K. Figure S3: Estimation of four parameters (kinetic coefficient *β*_0_, interfacial energy *σ*_0_, and their anisotropy parameters, *ε_k_* and *ε_c_*) using observation data of molecular dynamics (MD) simulation at 1530 K.

## Appendix A. Mathematical Expression of Ensemble Kalman Filter

In the EnKF, system and observation models are defined as

(A1) $$x_{t}=f_{t}\left(x_{t-1}\right)+v_{t}$$

(A2) $$y_{t}=H_{t}x_{t}+w_{t}$$

(A3) $$w_{t}\sim N\left(0,R_{t}\right)$$

***w****_t_* is given as Gaussian distribution with zero mean and a covariance matrix of ***R****_t_*. In the prediction step, time evolution of states vector is calculated as

(A4) $$x_{t|t-1}^{\left(i\right)}=f_{t}\left(x_{t-1|t-1}^{\left(i\right)}\right)+v_{t}^{\left(i\right)}$$

where superscript (*i*) represents *i*th state in *N* ensembles. Subscript *a*|*b* represents the state vector of time *a*, which is filtered at time *b*. Probability distribution function after the prediction step is given as

(A5) $$p\left(x_{t}|y_{1:t-1}\right)≅\frac{1}{N}\overset{N}{\underset{i=1}{\sum }}\delta \left(x_{t}-x_{t|t-1}^{\left(i\right)}\right)$$

where *δ* is the Dirac delta function, and ***y***_1:*t*__−1_ represents observational data from time *t* = 1 to time *t* − 1. In the filtering step, states vector is updated as [44,52]:

(A6) $$x_{t|t}^{\left(i\right)}=x_{t|t-1}^{\left(i\right)}+K_{t}\left(y_{t}+{\tilde{w}}_{t}^{\left(i\right)}-H_{t}x_{t-1|t-1}^{\left(i\right)}\right)$$

***K****_t_* is ensemble approximation of Kalman gain, given as

(A7) $$K_{t}=V_{t|t-1}H_{t}^{T}{\left(H_{t}V_{t|t-1}H_{t}^{T}+R_{t}\right)}^{-1}$$

$V_{t|t-1}$ and ***R****_t_* are sample covariance matrixes of state vector and observation error, given as:

(A8) $$V_{t|t-1}=\frac{1}{N-1}\Sigma_{i=1}^{N}{\tilde{x}}_{t|t-1}^{\left(i\right)}{\left({\tilde{x}}_{t|t-1}^{\left(i\right)}\right)}^{T}$$

(A9) $${\tilde{x}}_{t|t-1}^{\left(i\right)}=x_{t|t-1}^{\left(i\right)}-\frac{1}{N}\Sigma_{i=1}^{N}x_{t|t-1}^{\left(i\right)}$$

(A10) $$R_{t}=\frac{1}{N-1}\Sigma_{i=1}^{N}{\tilde{w}}_{t}^{\left(i\right)}{\left({\tilde{w}}_{t}^{\left(i\right)}\right)}^{T}$$

(A11) $${\tilde{w}}_{t}^{\left(i\right)}=w_{t}^{\left(i\right)}-\frac{1}{N}\Sigma_{i=1}^{N}w_{t}^{\left(i\right)}$$

Probability distribution function after the filtering step is given as

(A12) $$p\left(x_{t}|y_{1:t}\right)≅\frac{1}{N}\overset{N}{\underset{i=1}{\sum }}\delta \left(x_{t}-x_{t|t}^{\left(i\right)}\right)$$

## References

[1] Kobayashi R. (1993). Modeling and numerical simulations of dendritic crystal growth. Phys. D.

[2] Asta M., Beckermann C., Karma A., Kurz W., Napolitano R., Plapp M., Purdy G., Rappaz M., Trivedi R. (2009). Solidification microstructures and solid-state parallels: Recent developments, future directions. Acta Mater..

[3] Takaki T. (2014). Phase-field modeling and simulations of dendrite growth. ISIJ Int..

[4] Ohno M. (2020). Quantitative Phase-field Modeling and Simulations of Solidification Microstructures. ISIJ Int..

[5] Boettinger W.J., Warren J.A., Beckermann C., Karma A. (2002). Phase field simulation of solidification. Annu. Rev. Mater. Res..

[6] Takaki T., Sakane S., Ohno M., Shibuta Y., Aoki T., Gandin C.-A. (2018). Competitive grain growth during directional solidification of a polycrystalline binary alloy: Three-dimensional large-scale phase-field study. Materialia.

[7] Suwa Y., Saito Y., Onodera H. (2007). Three-dimensional phase field simulation of the effect of anisotropy in grain-boundary mobility on growth kinetics and morphology of grain structure. Comput. Mater. Sci..

[8] Kim S.G., Kim D.L., Kim W.T., Park Y.B. (2006). Computer simulations of two-dimensional and three-dimensional ideal grain growth. Phys. Rev. E.

[9] Miyoshi E., Takaki T., Ohno M., Shibuta Y., Sakane S., Shimokawabe T., Aoki T. (2017). Ultra-large-scale phase-field simulation study of ideal grain growth. NPJ Comput. Mater..

[10] Takaki T., Hisakuni Y., Hirouchi T., Yamanaka A., Tomita Y. (2009). Multi-phase-field simulations for dynamic recrystallization. Comput. Mater. Sci..

[11] Militzer M., Mecozzi M., Sietsma J., van der Zwaag S. (2006). Three-dimensional phase field modelling of the austenite-to-ferrite transformation. Acta Mater..

[12] Yamanaka A., Takaki T., Tomita Y. (2010). Elastoplastic phase-field simulation of martensitic transformation with plastic deformation in polycrystal. Int. J. Mech. Sci..

[13] Turnbull D. (1950). Formation of crystal nuclei in liquid metals. J. Appl. Phys..

[14] Waseda Y., Miller W.A. (1978). Calculation of the crystal-melt interfacial free energy from experimental radial distribution function data. Trans. JIM.

[15] Gündüz M., Hunt J.D. (1985). The measurement of solid-liquid surface energies in the Al-Cu, Al-Si and Pb-Sn systems. Acta Metall..

[16] Broughton J.Q., Gilmer G.H. (1986). Molecular dynamics investigation of the crystal–fluid interface. VI. Excess surface free energies of crystal–liquid systems. J. Chem. Phys..

[17] Hoyt J., Asta M., Haxhimali T., Karma A., Napolitano R., Trivedi R., Laird B.B., Morris J. (2004). Crystal–melt interfaces and solidification morphologies in metals and alloys. MRS Bull..

[18] Hoyt J.J., Asta M., Karma A. (2001). Method for computing the anisotropy of the solid-liquid interfacial free energy. Phys. Rev. Lett..

[19] Morris J.R., Song X. (2003). The anisotropic free energy of the Lennard-Jones crystal-melt interface. J. Chem. Phys..

[20] Watanabe Y., Shibuta Y., Suzuki T. (2010). Molecular dynamics study of thermodynamic and kinetic properties of solid-liquid interface for bcc iron. ISIJ Int..

[21] Shibuta Y., Ohno M., Takaki T. (2005). Solidification in a Supercomputer: From Crystal Nuclei to Dendrite Assemblages. JOM.

[22] Saidi P., Freitas R., Frolov T., Asta M., Hoyt J.J. (2017). Free energy of steps at faceted (1 1 1) solid-liquid interfaces in the Si-Al system calculated using capillary fluctuation method. Comput. Mater. Sci..

[23] Qi C., Xu B., Kong L.T., Li J.F. (2017). Solid-liquid interfacial free energy and its anisotropy in the Cu-Ni binary system investigated by molecular dynamics simulations. J. Alloys Compd..

[24] Ueno K., Shibuta Y. (2020). Solid-liquid interfacial energy for Fe-Cr alloy under temperature gradient from molecular dynamics simulation. ISIJ Int..

[25] Ueno K., Shibuta Y. (2019). Composition dependence of solid-liquid interfacial energy of Fe-Cr binary alloy from molecular dynamics simulations. Comput. Mater. Sci..

[26] Kavousi S., Novak B.R., Baskes M.I., Zaeem M.A., Moldovan D. (2020). Modified embedded-atom method potential for high-temperature crystal-melt properties of Ti–Ni alloys and its application to phase field simulation of solidification. Model. Simul. Mater. Sci. Eng..

[27] Bai X.-M., Li M. (2006). Calculation of solid-liquid interfacial free energy: A classical nucleation theory based approach. J. Chem. Phys..

[28] Sun Y., Zhang F., Song H., Mendelev M.I., Wang C.-Z., Ho K.-M. (2018). Temperature dependence of the solid-liquid interface free energy of Ni and Al from molecular dynamics simulation of nucleation. J. Chem. Phys..

[29] Hoyt J.J., Asta M., Karma A. (2003). Atomistic and continuum modeling of dendritic solidification. Mater. Sci. Eng. R.

[30] Shibuta Y., Ohno M., Takaki T. (2018). Advent of cross-scale modeling: High-performance computing of solidification and grain growth. Adv. Theory Simul..

[31] Spapen F. (1975). A structural model for the solid-liquid interface in monatomic systems. Acta Metall..

[32] Gránásy L., Tegze M., Ludwig A. (1991). Solid-liquid interfacial free energy. Mater. Sci. Eng. A.

[33] Spaepen F. (1994). The temperature dependence of the crystal-melt interfacial tension: A simple model. Mater. Sci. Eng. A.

[34] Davidchack R.L., Laird B.B. (2003). Direct calculation of the crystal–melt interfacial free energies for continuous potentials: Application to the Lennard-Jones system. J. Chem. Phys..

[35] Laird B.B., Davidchack R.L. (2005). Direct calculation of the crystal-melt interfacial free energy via molecular dynamics computer simulation. J. Phys. Chem. B.

[36] Jian Z., Li N., Zhu M., Chen J., Chang F., Jie W. (2012). Temperature dependence of the crystal–melt interfacial energy of metals. Acta Mater..

[37] Baidakov V.G., Tipeev A.O. (2012). Crystal nucleation and the solid–liquid interfacial free energy. J. Chem. Phys..

[38] Lippmann S., Jung I.-H., Paliwal M., Rettenmayr M. (2016). Modelling temperature and concentration dependent solid/liquid interfacial energies. Philos. Mag..

[39] Tognato R. (1992). On the temperature dependence of solid-liquid interfacial free energies per unit area. Phase Transit..

[40] Mondal K., Kumar A., Gupta G., Murty B.S. (2009). Temperature and structure dependency of solid–liquid interfacial energy. Acta Mater..

[41] Wu D.T., Gránásy L., Spaepen F. (2004). Nucleation and the solid–liquid interfacial free energy. MRS Bull..

[42] Hoyt J.J., Raman S.M., Asta M. (2018). Unusual temperature dependence of the solid-liquid interfacial free energy in the Cu-Zr system. Comput. Mater. Sci..

[43] Wang L., Hoyt J.J., Wang N., Provatas N., Sinclair C.W. (2020). Controlling solid-liquid interfacial energy anisotropy through the isotropic liquid. Nat. Commun..

[44] Wang L., Hoyt J.J. (2021). Layering misalignment and negative temperature dependence of interfacial free energy of B2-liquid interfaces in a glass forming system. Acta Mater..

[45] Cheng B., Tribello G.A., Ceriotti M. (2015). Solid-liquid interfacial free energy out of equilibrium. Phys. Rev. B.

[46] Ohno M., Oka Y., Sakane S., Shibuta Y., Takaki T. (2020). Bayesian inference of solid-liquid interfacial properties out of equilibrium. Phys. Rev. E.

[47] Lahoz W., Khattatov B., Menard R. (2010). Data Assmilation—Making Sense of Observations.

[48] Ghil M., Malanotte-Rizzoli P. (1991). Data assimilation in meteorology and oceanography. Adv. Geophys..

[49] Sasaki K., Yamanaka A., Ito S., Nagao H. (2018). Data assimilation for phase-field models based on the ensemble Kalman filter. Comput. Mater. Sci..

[50] Yamanaka A., Maeda Y., Sasaki K. (2019). Ensemble Kalman filter-based data assimilation for three-dimensional multi-phase-field model: Estimation of anisotropic grain boundary properties. Mater. Design.

[51] Oka Y., Ohno M. (2020). Parameter estimation for heat transfer analysis during casting processes based on ensemble Kalman filter. Int. J. Heat Mass Trans..

[52] Natsume Y., Oka Y., Ogawa J., Ohno M. (2020). Estimation of time-dependent heat transfer coefficient in unidirectional casting using a numerical model coupled with solidification analysis and data assimilation. Int. J. Heat Mass Trans..

[53] Shiraiwa T., Enoki M., Goto S., Hiraide T. (2020). Data Assimilation in the Welding Process for Analysis of Weld Toe Geometry and Heat Source Model. ISIJ Int..

[54] Shiraiwa T., Takahashi H., Enoki M. (2020). Acoustic emission analysis during fatigue crack propagation by Bayesian statistical modeling. Mater. Sci. Eng. A.

[55] Bragard J., Karma A., Lee Y.H., Plapp M. (2002). Linking phase-field and atomistic simulations to model dendritic solidification in highly undercooled melts. Interface Sci..

[56] Kalman R.E. (1960). A new approach to linear filtering and prediction problems. J. Basic. Eng..

[57] Evensen G. (1994). Sequential data assimilation with a nonlinear quasi-geostrophic model using Monte Carlo methods to forecast error statistics. J. Geophys. Res..

[58] Evensen G. (2003). The ensemble Kalman filter: Theoretical formulation and practical implementation. Ocean. Dyn..

[59] Doucet A., Godsill S., Andrieu C. (2000). On sequential Monte Carlo sampling methods for Bayesian filtering. Stat. Comput..

[60] Orihara S., Shibuta Y., Mohri T. (2020). Molecular dynamics simulation of nucleation from undercooled melt of nickel-aluminum alloy and discussion on polymorphism in nucleation. Mater. Trans..

[61] Plimpton S.J. (1995). Fast parallel algorithms for short-range molecular dynamics. J. Comput. Phys..

[62] Purja Pun G.P., Mishin Y. (2009). Development of an interatomic potential for the Ni-Al system. Philos. Mag..

[63] Mishin Y. (2004). Atomistic modeling of the γ and γ’-phases of the Ni–Al system. Acta Mater..

[64] Sun D.Y., Asta M., Hoyt J.J. (2004). Crystal-melt interfacial free energies and mobilities in fcc and bcc Fe. Phys. Rev. B.

[65] Nose S. (1984). A unified formulation of the constant temperature molecular dynamics methods. J. Chem. Phys..

[66] Hoover W.G. (1985). Canonical dynamics: Equilibrium phase-space distributions. Phys. Rev. A.

[67] Shibuta Y., Oguchi K., Ohno M. (2014). Million-atom molecular dynamics simulation on spontaneous evolution of anisotropy in solid nucleus during solidification of iron. Scr. Mater..

[68] Schneider T., Stoll E. (1978). Molecular-dynamics study of a three-dimensional one-component model for distortive phase transitions. Phys. Rev. B.

[69] Shibuta Y., Takamoto S., Suzuki T. (2008). A molecular dynamics study of the energy and structure of the symmetric tilt boundary of iron. ISIJ Int..

[70] Shibuta Y. (2019). Estimation of thermodynamic and interfacial parameters of metallic materials by molecular dynamics simulations. Mater. Trans..

[71] Larsen P.M., Schmidt S., Schiøtz J. (2016). Robust structural identification via polyhedral template matching. Model. Simul. Mater. Sci. Eng..

[72] Stukowski A. (2020). Visualization and analysis of atomistic simulation data with OVITO-the Open Visualization Tool. Model. Simul. Mater. Sci. Eng..

[73] Miyoshi E., Takaki T., Shibuta Y., Ohno M. (2018). Bridging molecular dynamics and phase-field methods for grain growth prediction. Comput. Mater. Sci..

[74] Shibuta Y., Sakane S., Miyoshi E., Takaki T., Ohno M. (2019). Micrometer-scale molecular dynamics simulation of microstructure formation linked with multi-phase-field simulation in same space scale. Model. Simul. Mater. Sci. Eng..

[75] Turnbull D., Cech R.E. (1950). Microscopic observation of the solidification of small metal droplets. J. Appl. Phys..

[76] Vinet B., Magnusson L., Fredriksson H., Desre P.J. (2002). Correlations between surface and interface energies with respect to crystal nucleation. J. Colloid Interf. Sci..

[77] Hoyt J.J., Sadigh B., Asta M., Foiles S.M. (1999). Kinetic phase field parameters for the Cu–Ni system derived from atomistic computations. Acta Mater..

