# Supplementary material for: Bayesian Data Assimilation of Temperature Dependence of Solid–Liquid Interfacial Properties of Nickel
Source: Nanomaterials (Basel). 2021 Sep 6;11(9):2308. doi: 10.3390/nano11092308 (PMC8471389; doi:10.3390/nano11092308)
Supplement: Supplementary file 1 [file nanomaterials-11-02308-s001.zip › nanomaterials-1355779-supplementary.pdf]

nanomaterials

MDPI

Supplementary Materials

Bayesian Data Assimilation of Temperature Dependence of Solid–Liquid Interfacial Properties of Nickel

Yuhi Nagatsuma [1], Munekazu Ohno [2], Tomohiro Takaki [3] and Yasushi Shibuta [1,*]

[1] Department of Materials Engineering, The University of Tokyo, Tokyo 113-8656, Japan; nagatsuma-yuhi565@g.ecc.u-tokyo.ac.jp
[2] Division of Materials Science and Engineering, Faculty of Engineering, Hokkaido University, Sapporo 060-8628, Japan; mohno@eng.hokudai.ac.jp
[3] Faculty of Mechanical Engineering, Kyoto Institute of Technology, Kyoto 606-8585, Japan; takaki@kit.ac.jp
* Correspondence: shibuta@material.t.u-tokyo.ac.jp

Table S1. Representative properties of the EAM potential for Ni employed in this study [1,2].

Property	Experiment	EAM
Lattice constant, a_0 (nm)	0.352	0.352
Cohesive energy, e_0 (eV)	−4.45	−4.45
Elastic constants (GPa)		
B	181.0	181.0
c_{11}	246.5	241.3
c_{12}	147.3	150.8
c_{44}	124.7	127.3
Vacancy formation energy, E_v^f (eV)	1.60	1.57
Intrinsic stacking fault energy, γ_{SF} (mJ/m^2)	125	134
Symmetrical twin boundary energy, γ_T (mJ/m^2)	43	68
Surface energies (mJ/m^2)		
(110)	2280* (average)	2087
(100)	2280* (average)	1936
(111)	2280* (average)	1759

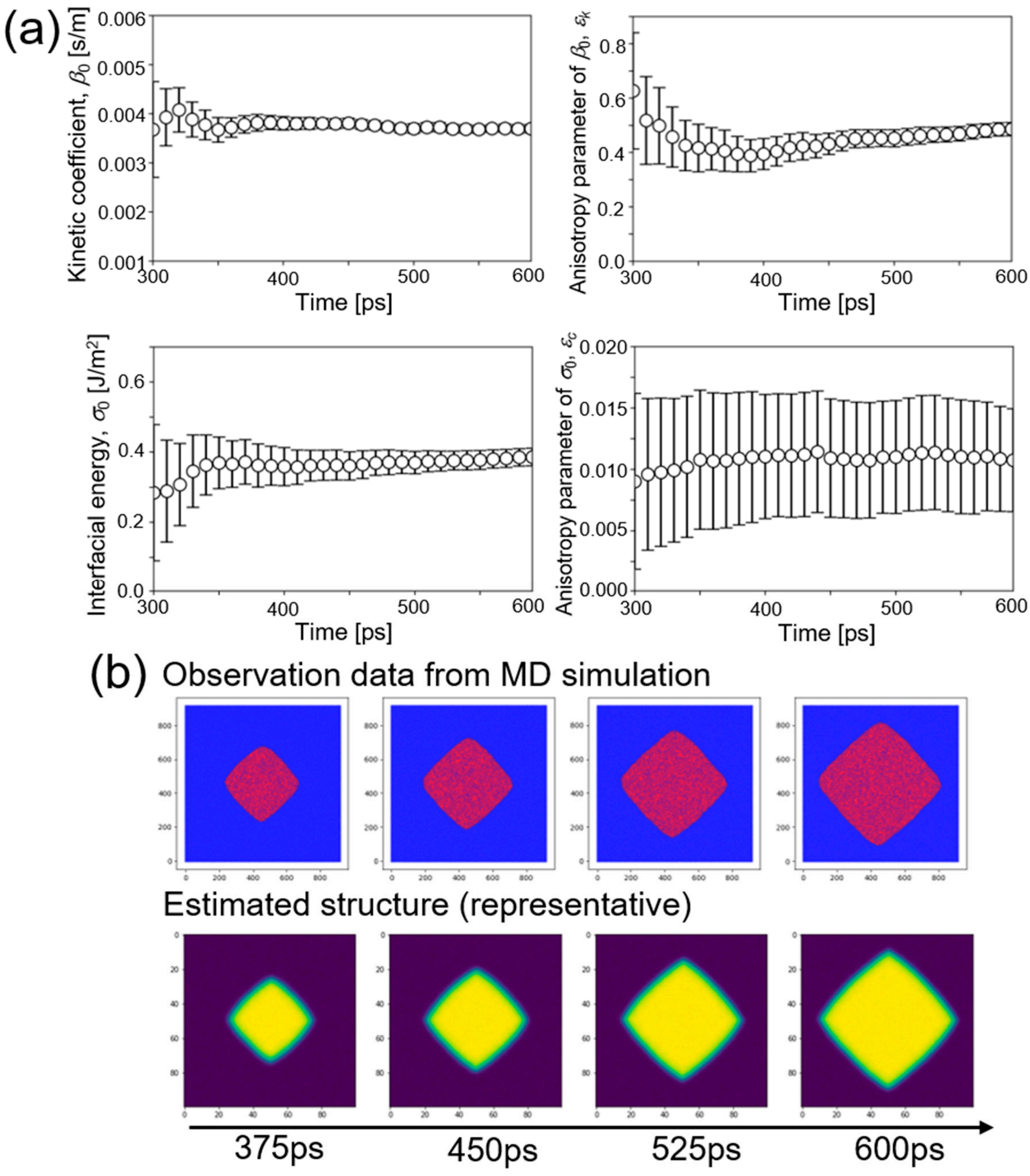

Figure S1. Estimation of four parameters (kinetic coefficient β_0, interfacial energy σ_0, and their anisotropy parameters ε_k and ε_c using observation data of molecular dynamics (MD) simulation at 1455 K. (**a**) Time changes of the estimated values of four parameters, β_0, σ_0, ε_k and ε_c. (**b**) Snapshots of observation data from MD simulation and representative result of estimated structure.

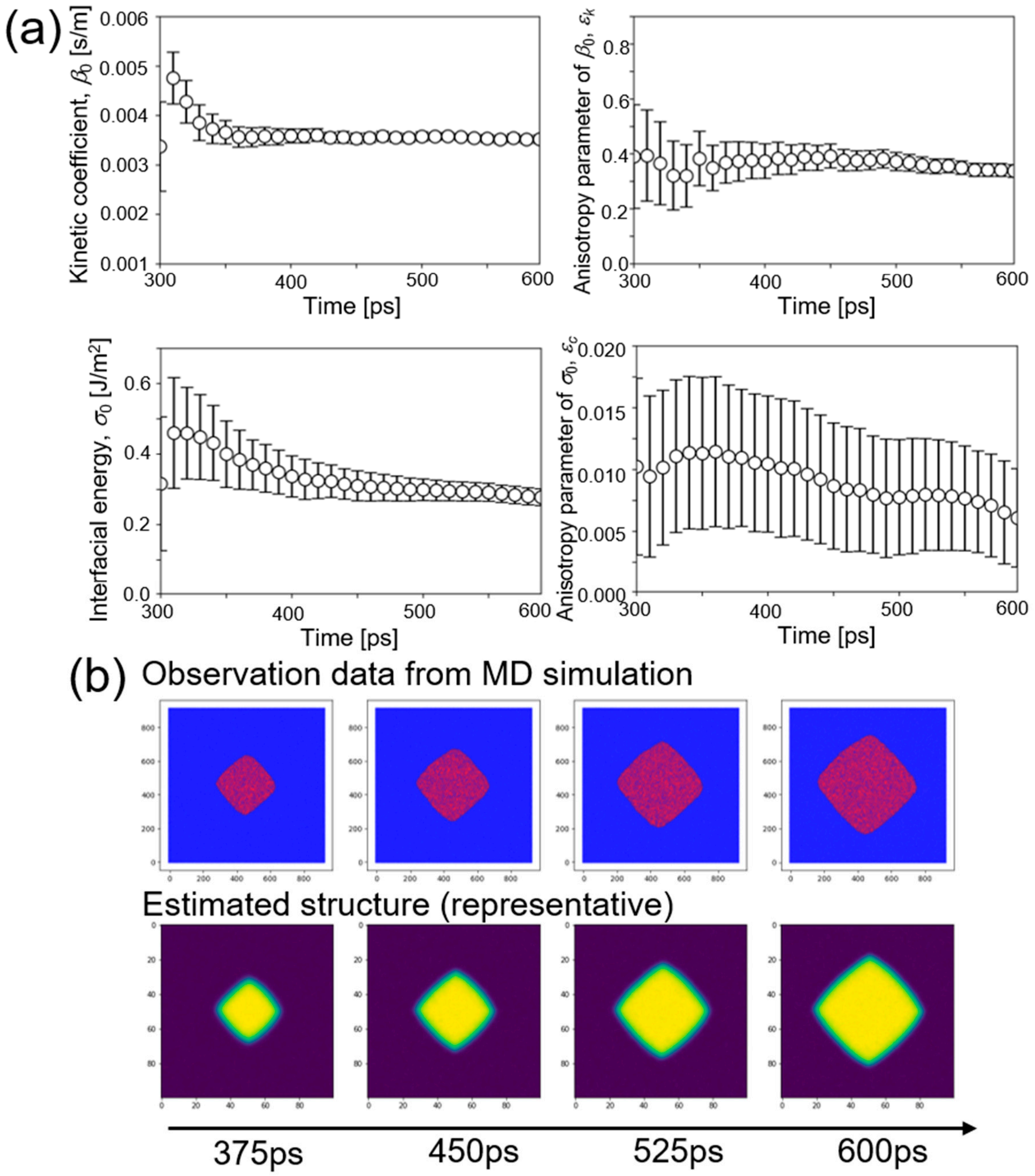

Figure S2. Estimation of four parameters (kinetic coefficient β_0, interfacial energy σ_0, and their anisotropy parameters ε_k and ε_c using observation data of molecular dynamics (MD) simulation at 1505 K. (**a**) Time changes of the estimated values of four parameters, β_0, σ_0, ε_k and ε_c. (**b**) Snapshots of observation data from MD simulation and representative result of estimated structure.

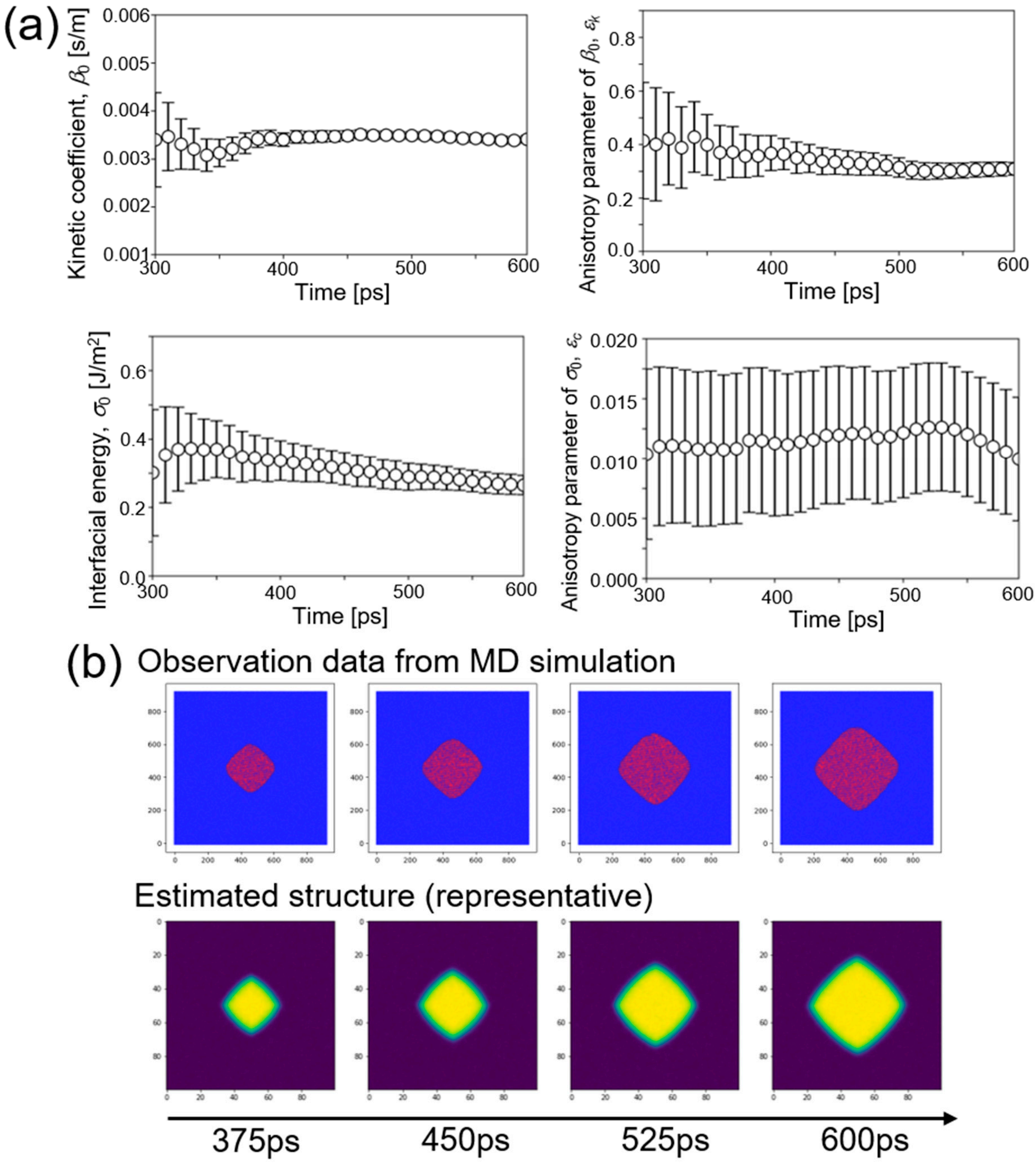

Figure S3. Estimation of four parameters (kinetic coefficient β_0, interfacial energy σ_0, and their anisotropy parameters ε_k and ε_c using observation data of molecular dynamics (MD) simulation at 1530 K. (**a**) Time changes of the estimated values of four parameters, β_0, σ_0, ε_k and ε_c. (**b**) Snapshots of observation data from MD simulation and representative result of estimated structure.

References

1. Purja Pun, G.P.; Mishin, Y. Development of an interatomic potential for the Ni-Al system. *Philos. Mag.* **2009**, *89*, 3245–3267.
2. Mishin, Y. Atomistic modeling of the γ and γ′-phases of the Ni–Al system. *Acta Mater.* **2004**, *52*, 1451–1467.
